# Magma transfer at Campi Flegrei caldera (Italy) before the 1538 AD eruption

**DOI:** 10.1038/srep32245

**Published:** 2016-08-25

**Authors:** Mauro A. Di Vito, Valerio Acocella, Giuseppe Aiello, Diana Barra, Maurizio Battaglia, Antonio Carandente, Carlo Del Gaudio, Sandro de Vita, Giovanni P. Ricciardi, Ciro Ricco, Roberto Scandone, Filippo Terrasi

**Affiliations:** 1 Istituto Nazionale di Geofisica e Vulcanologia, Sezione di Napoli Osservatorio Vesuviano, via Diocleziano 328, 80124 Napoli, Italy; 2 Dipartimento di Scienze Università Roma Tre, Italy; 3 Dipartimento di Scienze della Terra, dell’Ambiente e delle Risorse, Università degli Studi di Napoli Federico II, Italy; 4 Dipartimento di Scienze della Terra, Sapienza, Roma, Italy; 5 Volcano Science Center, US Geological Survey, Menlo Park, CA 94025, USA; 6 Dipartimento di Matematica e Fisica, Seconda Università di Napoli, Italy

## Abstract

Calderas are collapse structures related to the emptying of magmatic reservoirs, often associated with large eruptions from long-lived magmatic systems. Understanding how magma is transferred from a magma reservoir to the surface before eruptions is a major challenge. Here we exploit the historical, archaeological and geological record of Campi Flegrei caldera to estimate the surface deformation preceding the Monte Nuovo eruption and investigate the shallow magma transfer. Our data suggest a progressive magma accumulation from ~1251 to 1536 in a 4.6 ± 0.9 km deep source below the caldera centre, and its transfer, between 1536 and 1538, to a 3.8 ± 0.6 km deep magmatic source ~4 km NW of the caldera centre, below Monte Nuovo; this peripheral source fed the eruption through a shallower source, 0.4 ± 0.3 km deep. This is the first reconstruction of pre-eruptive magma transfer at Campi Flegrei and corroborates the existence of a stationary oblate source, below the caldera centre, that has been feeding lateral eruptions for the last ~5 ka. Our results suggest: 1) repeated emplacement of magma through intrusions below the caldera centre; 2) occasional lateral transfer of magma feeding non-central eruptions within the caldera. Comparison with historical unrest at calderas worldwide suggests that this behavior is common.

Defining and understanding the shallow transfer of magma at volcanoes is crucial to forecast eruptions, possibly the ultimate goal of volcanology. This is particularly challenging at felsic calderas experiencing unrest, which typically includes significant changes in seismicity, deformation and degassing rates. In fact, caldera unrest is particularly frequent, affects wide areas and its evidence is often complicated by the presence of a hydrothermal system: as a result, forecasting any eruption and vent-opening sites within an existing caldera is very difficult^1^.

In historical times only two felsic restless calderas have erupted: Campi Flegrei and Rabaul^2^. Campi Flegrei, in the densely inhabited metropolitan area of Naples (Italy), is commonly considered one of the most dangerous active volcanic systems. Campi Flegrei is a ~12 km wide depression hosting two nested calderas formed during the eruptions of the Campanian Ignimbrite (~39 ka) and the Neapolitan Yellow Tuff (~15 ka) (Fig. 1; refs^ 3^, ^4^, ^5^, ^6^). In the last ~5 ka, resurgence^7^ [references therein], with uplift >60 m close to the central part of the caldera (the Pozzuoli area), was accompanied by volcanism of the “III epoch” of activity (~4.8 to ~3.8 ka; ref. 7). After ~3 ka of quiescence, several decades of increasing seismicity and uplift preceded the last eruption at Monte Nuovo in 1538^7^,^8^,^9^. The most recent activity culminated in four unrest episodes between 1950–1952, 1969–1972, 1982–1984 and 2005-Present, with uplift at Pozzuoli of ~0.7, ~1.7, ~1.8 and ~0.3 m, respectively^10^,^11^; the present unrest episode has been interpreted as being magma-driven^12^,^13^. These unrest episodes are considered the most evident expression of a longer-term (centuries or more) restless activity^4^,^10^. The post-1980 deformation largely results from a magmatic oblate or sill-like source at ~4 km depth below Pozzuoli (e.g.^12^ and references therein^13^); however, an important role for the hydrothermal system has been also proposed^14^ [references therein].

Despite the restless activity of Campi Flegrei, the recent unrest episodes did not culminate in eruption, so that any possibility to define the pre-eruptive shallow transfer of magma (that is, from the magma reservoir to the surface) at Campi Flegrei remains elusive. Indeed, this definition is a crucial step in order to identify and understand pre-eruptive processes, and thus to make any forecast. To fill this gap, we focused on the last eruption of 1538, reconstructing its pre-eruptive deformation pattern. For this, we exploited the unique historical, archaeological, geological and long-term geodetic record of the caldera to carefully determine the height variations (and related errors) of 20 selected sites along its coastline (Fig. 1 and Supplementary Table S1). The details of this complex and multidisciplinary approach are provided in the Methods and in Supplementary Information sections.

In this paper a completely original data set is provided on the height variations within the Campi Flegrei caldera in the last 2000 years, with the only exception for the previously known vertical displacements at site 30 (Fig. 1). While our collected data span the last 2000 years of evolution of the caldera, in this study we focus only on the deformation occurring ~300 years before the 1538 eruption, when the centuries-long subsidence of the caldera reversed into uplift.

## Results

### Elevation changes within the caldera

**Figure 1:**
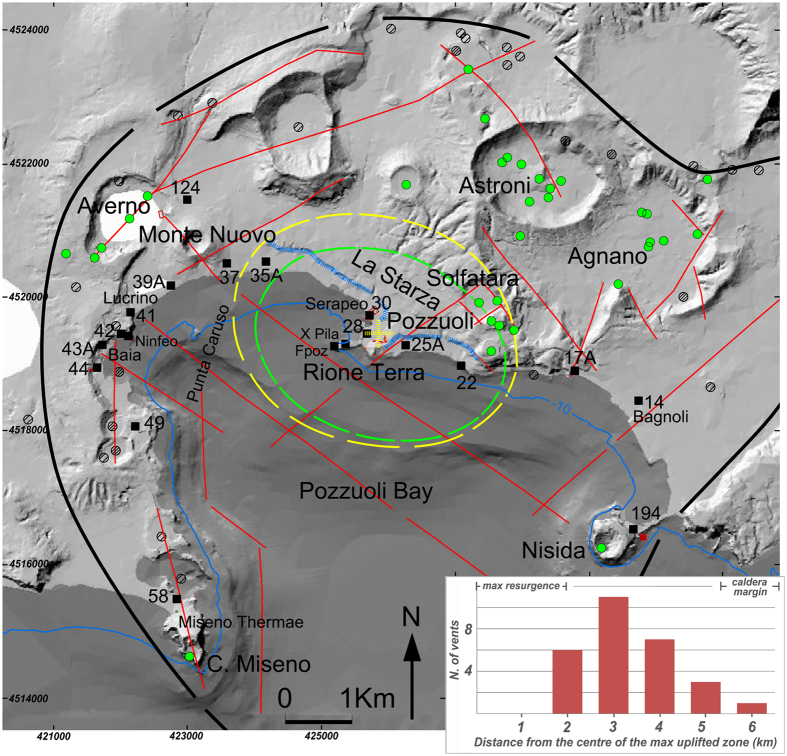
Morphological and structural sketch map of the Campi Flegrei caldera. Circles: vents of the I epoch (15-9.5 ka BP; shaded), II epoch (8.6-8.2 ka BP; densely shaded) and III epoch (4.8-3.8 ka BP; green) of volcanic activity. Red: main faults and fractures; black line: Neapolitan Yellow Tuff caldera; yellow ellipse and cross: maximum uplifted sector and caldera centre in the last 5 ka; green ellipse: projection of the quasi-horizontal source (pressurized triaxial ellipsoid ~4000 m deep) of recent ground deformation^12^. Black squares: selected benchmarks and relative number (names in Supplementary Table S1). Thick blue line: cliff of the La Starza uplifted marine terrace. Thinner blue line: 10 m bsl isobath. Inset: distance of the eruptive vents active in the last 5 ka from the centre of the maximum uplifted zone in the same period. *Digital Terrain Model by INGV-Osservatorio Vesuviano.*

The integrated analysis of geomorphological, sedimentological, paleontological, archaeological and historical data allowed a detailed and quantitative reconstruction of the evolution of the ground displacements predating the Mt. Nuovo eruption along the coastline of the Pozzuoli Bay (Fig. 1). A representative example of the multidisciplinary procedure adopted for such a detailed description of the historical elevation changes for the Capo Miseno area is included in the supplementary material.

**Figure 2:**
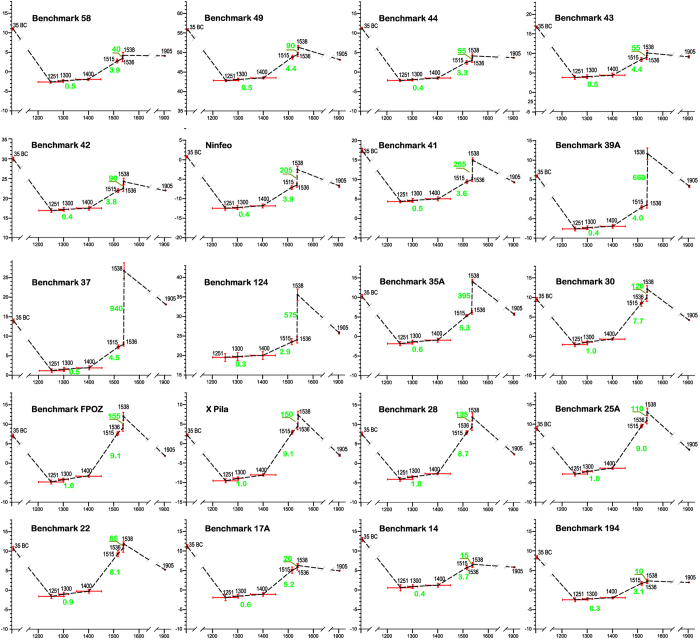
Reconstruction of the elevation (m above the sea level in 1905 – first leveling by Istituto Geografico Militare) through time at 20 selected sites within the Campi Flegrei caldera, obtained integrating geological, historical and archaeological data. The elevation is referred to the nearest benchmark or to archaeological structures. The uplift rate (cm/yr) calculated for the periods 1251–1400, 1400–1536 and 1536–1538 is reported in green. The location of the sites is reported in Fig. 1, from Miseno (benchmark 58) to Nisida (benchmark 194). Error bars are in red. The diagrams highlight the difference in elevation changes between the benchmarks close to the caldera centre and those near Monte Nuovo. During 1251–1400 the caldera floor underwent a minor uplift, with maximum values in the Pozzuoli area. From 1400 to 1536 a general and sharp increase of the uplift rate still culminates in the Pozzuoli area. Immediately before the eruption (1536–1538) the uplift reaches the highest rate in the area of opening of the future vent (Mt. Nuovo). *Data by the authors.*

The general results of our analysis are summarized in Fig. 2, which shows the historical elevation changes, from 35 BC to Present, at the sites along the coastline of Pozzuoli Bay. These data show that in 35 BC the coastline extended outward into what is now the Pozzuoli Bay. However, since then all the area started to be affected by a quick subsidence^4^, which resulted in progressive submersion of the coastline until 1251. The amount of subsidence in the investigated area varies from place to place (Fig. 2) and is well documented by the presence of geomorphological, sedimentological and paleontological indicators. A subsequent progressive emersion of the area started during the 13^th^ century, as suggested by historical and urban planning sources, archaeological evidence and geological data (Supplementary Tables S1 and S2). The lower time limit for the caldera uplift is given by historical documents describing the Pozzuoli promontory as an island in 1251^15^,^16^, whereas at the end of the 13^th^ and beginning of the 14^th^ century the previously submerged area around the promontory is reported as the location of three new churches. Moreover, coeval urban studies testify to the expansion of Pozzuoli on new land formed by the coastline regression, confirming the onset of a long-term uplift^15^,^16^. Since sea-level variation in the last 2000 years has been on the order of 0.7 m on average^17^ [reference therein], the much larger (see below for values) emersion of the area from the 13^th^ to the 16^th^ century was mainly due to the ground uplift, with the maximum values recorded in the Pozzuoli area.

The uplift rate was quite low (0.3 to 1 cm/yr; Fig. 2; Supplementary Table S1) from the middle of the 13^th^ to the end of the 14^th^ century, and increased to 2.9 to 9.1 cm/yr from 1400 to 1536 (Fig. 2; Supplementary Table S1). During this latter time-span, all the coastal strip emerged in response to the generalized uplift of the caldera floor, whose maximum of 12.3 m has been recorded again in the Pozzuoli area (Fig. 2; Supplementary Table S1). Since the end of the 15^th^ century this uplift was accompanied by strong seismicity^9^. A new and stronger uplift, with a rate of 10 to 940 cm/yr (Fig. 2; Supplementary Table S1), followed the previous one between 1536–1538, reaching a maximum value of 18.8 m in the future vent-opening area (Mt. Nuovo; Fig. 2; Supplementary Table S1). This highest-rate uplift was accompanied by very intense seismicity, which affected all the Pozzuoli area and was felt also in the city of Naples^9^ [references therein]. Indeed, all the historical sources coeval to the eruption report an evident uplift accompanied by seismicity and opening of fractures in the vent area during the two days that preceded the eruption.

The error associated with the height estimate up to 1536 is relatively low (<1 m): we therefore use this year to distinguish the long-term caldera deformation from the short-term deformation preceding the eruption, from 1536 to 1538, reconstructed from several sources almost coeval with the eruption^18^,^19^,^20^,^21^,^22^. In the evaluation of the short-term deformation we do not take into account for the deformation occurred approximately two days before the onset of the eruptive activity, in order to exclude any contribution from the emplacement of the dike feeding the eruption; however, due to the very rapid deformation, the evaluation of the total amount of deformation in sites close to Monte Nuovo in the days before the eruption is affected by a larger error (up to 2.5 m).

After the eruption, all the recovered deformation data show a generalized renewal of the subsidence, with maximum values in the Pozzuoli area.

The nature of the data (inferred from historical and archaeological records) makes it difficult to precisely infer the amount and extent of horizontal deformation that accompanied the vertical deformation. However, records of ground tilt offer an additional constraint on the deformation field. To this aim, tilt changes between 1536 and 1538 have also been reconstructed (Supplementary Information and Supplementary Fig. S4).

### Modelling

**Figure 3:**
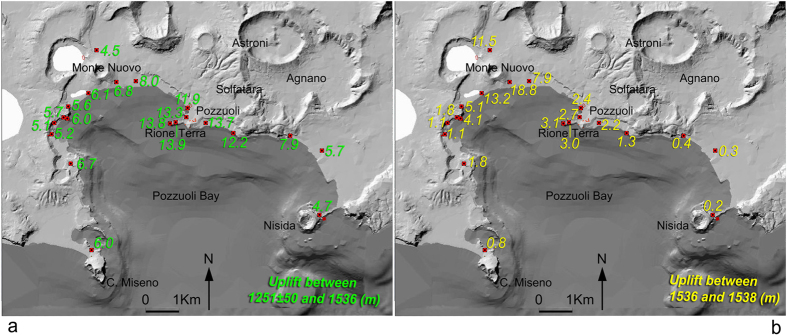
Distribution of the surface uplift preceding the Mt. Nuovo eruption. From 1251 to 1536 (**a**) the uplift affects the whole caldera, with a maximum in the Pozzuoli area. From 1536 to 1538 (**b**) the uplift is centred in the area of the future eruption (Monte Nuovo). *Digital Terrain Model by INGV-Osservatorio Vesuviano.*

Our reconstruction shows that between 1251 and 1536 a general cumulative uplift affected the inner caldera, with a maximum value of 14 m in Pozzuoli (Fig. 3a). The largest part of this deformation occurred between 1400 and 1536, with a maximum of about 12 m in Pozzuoli (Fig. 2 and supplementary Table S1). Therefore we used the data of this interval to model the source. The deformation from 1536 to 1538 (Fig. 3b) is centred on the area of the future eruption, with a maximum uplift of ~19 m; in this period the uplift along the eastern Pozzuoli Bay shows a trend similar to that of 1251–1536 (Supplementary Table S1).

We model the caldera’s crust as a homogenous, isotropic, elastic half space. Once we include the data uncertainty in our inversion model, our relatively simple approach allows us to get results that can be compared with those of more complex, numerical models. Therefore, while being aware of the limitations of our models (as occurring in most, if not all, models of volcano deformation), we also emphasize that our models provide a first-order analysis with an estimated and acceptable error^23^,^24^.

We did not attempt to model any contribution to the deformation field from the Campi Flegrei hydrothermal system or from structural discontinuities associated with the caldera. The nature, extent, and permeability of the pre-1538 hydrothermal system are highly uncertain, so any attempt to model the effect of magma accumulation on it would have introduced a set of largely unconstrained variables. Therefore, any modelling considering the role of the hydrothermal system would have just introduced a higher set of non-constrained variables. Similar considerations also hold in considering any pre-existing discontinuity in the modelling: our general knowledge and data on the subsurface of Campi Flegrei are still too limited to include a reliable and univocal analysis taking into account for pre-existing fractures.

Another limit of our analysis is that elastic deformation models have very similar near-field vertical deformation for a range of source geometries^25^. Resolution of the geometry of a source would require the inversion of 3D deformation data^24^ that cannot be inferred from the existing historical and archaeological records.

**Figure 4:**
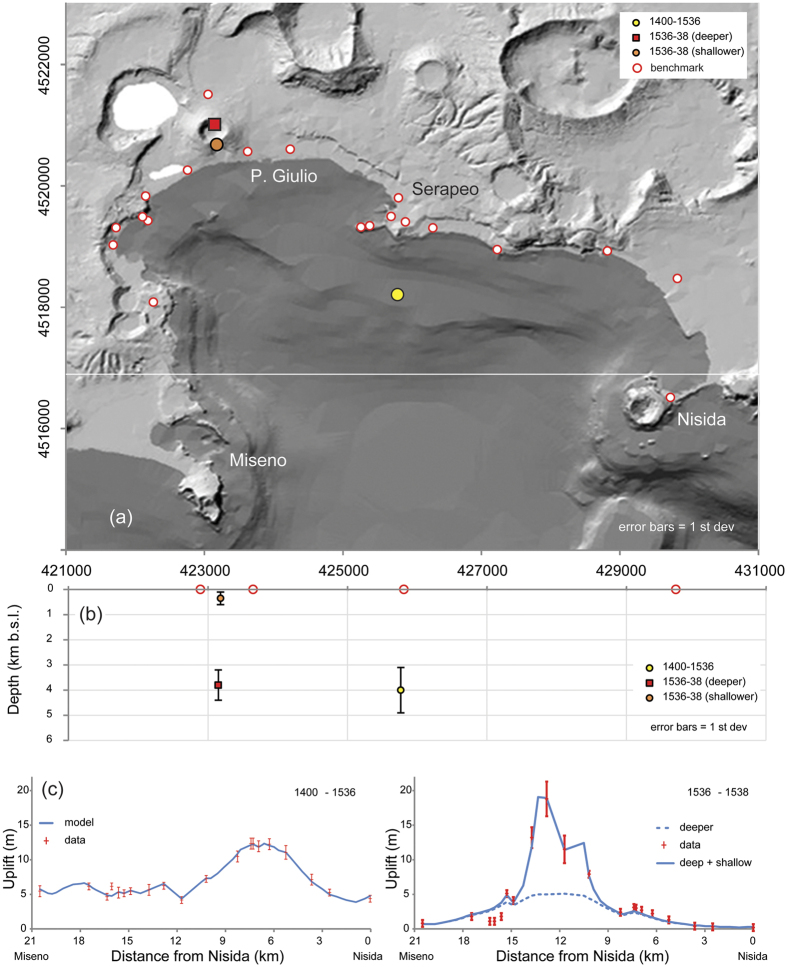
(**a**) Source location (1400–1536 and 1536–1538) – the white line gives the position of the section shown in (**b**); (**b**) Source depth (1400–1536 and 1536–1538); (**c**) deformation profiles and best-fit models. Details of the best-fit sources are available in Supplementary Table S3. A site by site comparison between the data and the best fit models is available in Supplementary Table 4. Error bars are 1 standard deviations. *Digital Terrain Model by INGV-Osservatorio Vesuviano.*

The inversion of the deformation data suggests that the best-fit source for the 1400–1536 uplift is a radially symmetrical intrusion (solutions from a sphere, spheroid or sill are very consistent regarding location, depth and volume change – see Supplementary Table 2) in the caldera centre (Fig. 4a), 1 to 2 km south of Pozzuoli at a depth between 3.9 and 5.5 km and with a volume change of 0.93 to 0.95 km^3^ (Fig. 4b). The best-fit solution for the 1536–1538 uplift and tilt change is a radially symmetrical intrusion lying 3.2 to 4.4 km beneath Monte Nuovo (Fig. 4a,b), with a volume change of 0.21 to 0.34 km^3^. A smaller, shallower intrusion beneath Monte Nuovo (depth 0.1 to 0.6 km) explains the large uplift at four sites close to Monte Nuovo (Fig. 4c). The volume change of this second source (0.03 to 0.05 km^3^) is consistent with the volume of pyroclastic ejecta from the eruption^7^. Again, solutions from a sphere, spheroid or sill show similar values for the location, depth and volume change of these two sources (Supplementary Table S3). A site by site comparison between the best-fit models and the deformation data is available in Fig. 4 and Supplementary Table S4.

## Discussion and Conclusions

Within error (see Fig. 4), the location and depth of the 1400–1536 source, below the caldera centre, are consistent with the source (defined by the inversion of geodetic data) responsible for most of the surface deformation since 1980^12^,^13^ and the previous unrest episodes^26^. The caldera centre also coincides with the maximum uplift of the resurgence (Fig. 1; ref. 7), suggesting that the magmatic source below has been active in the last ~5 ka and is thus long-lived and stable. Despite the central location and long-term persistence of the most uplifted area and the underlying magmatic source, the 1538 eruption occurred at the periphery of the resurgence and of the current magma reservoir^12^,^13^. The distribution of sin-resurgence vents (III epoch of caldera activity^7^) also focuses at a distance between 2 and 4 km from the caldera centre (Fig. 1), at the periphery of the current magma reservoir and resurgence, seldom reaching the inner caldera faults. All these features may be best explained by the activity of a magma reservoir at ~4 km depth resulting from the repeated emplacement of stacked tabular intrusions, or sills^27^, producing the cumulative uplift indicated by the resurgence. Stacked sills are the most common type of intrusion, as seen in eroded and exposed plutons^28^ [references therein] and suggested by geophysical data, also at deeper levels below Campi Flegrei^29^,^30^. In addition, sills are the easiest means to store magma in the upper crust, not requiring any tectonic contribution to create the space to emplace the magma^31^. Our modeling results support the conceptual model that sills occasionally propagate laterally as inclined sheets, transferring magma to shallower peripheral reservoirs and feeding the vents of the III epoch outside the Campi Flegrei caldera centre.

Alternatives to such a lateral transfer of magma are not supported by the available data. It may be argued that our data do not highlight any deflation of the central reservoir during the lateral transfer. However, in an open magmatic system, as during the last 15 ka at Campi Flegrei^32^ [references therein], the lateral migration of magma should be accompanied by the intrusion of new magma within the central reservoir, explaining the lack of any significant deflation; moreover, the temporal/spatial resolution of our geodetic record may not have detected any possible minor or short-term deflation. Similarly, any possible feeding of the eruption directly from a peripheral, deeper and hidden source bypassing the central reservoir is not supported by available petrological data, which show that the Monte Nuovo erupted products are among the most evolved of Campi Flegrei, resulting from the mixing between two distinct magmas, at least one of which is still present in the shallow (4–5 km deep) reservoir and has already been involved in other peripheral eruptions^32^,^33^,^34^.

**Figure 5:**
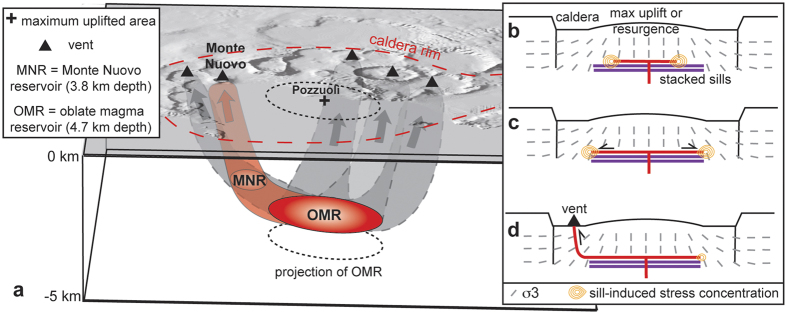
(**a**) Magma transfer below Campi Flegrei caldera before the Monte Nuovo eruption (orange); magma first propagated laterally from the oblate magma reservoir (OMR) at ~4.7 km depth below the caldera centre, feeding an eccentric reservoir (Monte Nuovo Reservoir, MNR) at ~3.8 km depth; from here, magma propagated vertically (orange) to feed a smaller and shallower reservoir (not shown) and then the Monte Nuovo eruption. The inferred paths (grey) for other representative eruptions of the last 5 ka are also reported; (**b–d**) General conceptual model for magma transfer below calderas, supported by all recent pre-eruptive unrest: a tabular intrusion, or sill, fed by a dike (red lines) forms at the top of a central magma chamber, possibly resulting from previous stacked tabular intrusions (purple lines) (**b**); the sill uplifts the caldera centre, where the previously stacked sills may have also promoted resurgence. Stresses (orange lines) focus at the tips of the sill, which propagates laterally following the minimum stress component σ_3_ (grey lines, schematically taken from ref. 24) controlled by the unloading promoted by the caldera depression (**c**); the sill eventually increases its dip and becomes a sub-vertical dike erupting to the side of the maximum uplifted area (black triangle) (**d**). Black arrows show the propagation direction of the intrusion. *Digital Terrain Model by INGV-Osservatorio Vesuviano.*

The available data suggest a conceptual model for the magmatic system of Campi Flegrei before the 1538 eruption and, more in general, in the last ~5 ka (Fig. 5), where magma propagation appears controlled by two processes:

1. The formation of sills along subhorizontal discontinuities below the caldera; geological, geophysical and modelling data show how the stresses focus at the tips of the sills, which propagate laterally, fracturing and intruding the host rock^35^ [references therein]. Analysis of historical and satellite geodetic data indicates the existence of a sill-like source at a depth of ~4 km that may explain the caldera deformation^12^.
2. The stresses promoted by the caldera unloading, expressed by the topographic (usually >100 m for Campi Flegrei), bathymetric (>50 m on average) and density (of 0.2–0.4 g/cm^3^; ref. 3) variations between the caldera centre and periphery^36^; these stresses are characterized by the progressive rotation of the minimum compression direction from sub-vertical below the caldera to sub-horizontal at the sides, as schematized in Fig. 5; these unloading stresses are estimated to be up to three orders of magnitude larger than those induced by the inflating central source^36^.

As a result of these two processes, the laterally propagating sills increase their dip, forming inclined sheets and then subvertical dikes feeding the eccentric vents. These stress variations may also explain the clustering of vents on the NE portion of the caldera, where there is a stronger topographic gradient^36^.

The pre-1538 uplift history also suggests that the emplacement of magma may have begun as early as ~300 years before the eruption, at a progressively increasing rate, varying over 3 orders of magnitude. It is likely that the estimated uplift rates, especially before 1536, reflect mean values of a discontinuous and incremental mode of magma emplacement below the caldera, with several episodes of inflation alternating with periods of stasis^37^. The more recent history of the caldera supports this incremental behaviour^10^ [references therein]. These periods of magma accumulation are also consistent with available petrological data, which suggest that pre-eruptive residence times at Campi Flegrei are in the order of ~100 years^38^,^39^,^40^.

Our model highlights the importance of considering pre-eruptive lateral magma propagation from the centre of Campi Flegrei caldera. Many calderas worldwide show different vent patterns. Outside the caldera, these include radial fissures along the flank of the caldera edifice and circumferential fissures along the outer caldera rim; these distal patterns of circumferential and radial dikes have been recently explained as due to the unloading due to the caldera depression, the depth to the magma reservoir and the density of the magma^36^ [references therein]. Within the caldera, vent patterns may have a wide variability, including scattered vents in a central or eccentric position and/or parallel fissures along regional structures [e.g.^41^]. Within this variability, some intra-caldera vent patterns appear more frequent. For example, most of the geodetically monitored (since the late 1980s) restless and erupting calderas show vents opening at the periphery of the area most uplifted during the unrest, often induced by sill-like magmatic sources. Examples include mafic and felsic calderas, such as Fernandina, Cerro Azul and Sierra Negra (Galapagos), Rabaul (Papua New Guinea), Aso and Usu (Japan) and Okmok (Aleutians)^42^ and references therein]. These examples suggest that the features found at Campi Flegrei may be relatively common at calderas worldwide, inasmuch as they have been documented at several well-monitored sites. Still, the longer-term geological record shows that some other calderas exhibit centred eruptive vents (e.g.^1^), suggesting that the proposed mechanism of lateral magma transfer does not apply in all cases. These deviations from our proposed conceptual model may be explained by several factors, such as the presence of a stronger regional extension, lack of a strong topographic caldera depression (both hindering lateral magma propagation), or the shape and size of the magmatic system.

The general applicability of our model to Campi Flegrei and several other calderas that have erupted since the late 1980s highlights lateral propagation as an important, perhaps predominant, process for magma transfer^43^. Our conceptual model explains the shallow lateral transfer of magma below Campi Flegrei in the last ~5 ka and, as supported by the available evidence of recent caldera unrest, may provide a key to forecasting the location of future eruptions at Campi Flegrei and calderas with similar behaviour.

## Methods

The reconstruction of the surface deformation (uplift and tilt) preceding the 1538 eruption is based on precise information on height variations at twenty sites along the Campi Flegrei coastline (Fig. 1) derived from archaeological, historical, geomorphological and stratigraphic evidence of sea-level persistence. Many archaeological remains of known age (Roman constructions and other later monuments and artefacts, such as harbour structures, roads, thermal baths, fishponds, churches and farms) permit the definition of the position of the coastline at the time of their construction. Moreover, paleontological, sedimentological and geochronological analyses of exposed and drilled sediment sequences from these twenty sites allowed the definition of their sedimentation environment, age and vertical motion. Vertical movements have been also inferred from the displacement of wave-cut notches and other erosional features, and by comparing the present depth of submerged structures and features (Fig. 1) with historical images and descriptions. Additional information has been obtained from chronicles and papers on the urban development of the town of Pozzuoli, and by comparing historical illustrations printed before and after the Monte Nuovo eruption. We also took into account for the thickness of the Monte Nuovo eruption deposits and the present height of their depositional surface, deduced from lithological, sedimentological and paleontological analysis of ~200 drill cores and logs (Supplementary Table S2 summarizes methods and related data, as well as the references used in the reconstruction of the ground deformation for each site and for selected periods). Finally, we considered information on sea-level variation during the past 2,000 years^17^.

The twenty measurement sites of vertical deformation were selected for their proximity (<100 m) to the benchmarks of the levelling network of the Osservatorio Vesuviano; this allowed us to subtract from our calculations the post-1905 (first levelling measurement by Istituto Geografico Militare, IGM^10^) vertical displacements. Ground displacement at the sites is thus referred to the nearest levelling benchmark. Supplementary Table S1 gives site coordinates, estimated ground-deformation, geological and archaeological evidence of sea-level variations and related references. Supplementary Information and Supplementary Figs S1, S2 and S3 provide an example of the multidisciplinary method used to reconstruct the ground movements at one representative site. The definition of the pre-Monte Nuovo ground deformation also allowed the mean ground tilt for the benchmarks (Supplementary Fig. S4) to be obtained.

Parameters of the caldera best-fitting deformation sources have been inferred by inverting uplift and tilt by using the dMODELS software package^44^. The software implements a number of analytical solutions for possible sources (sphere, spheroid, sill-like and opening crack/dike) in an elastic, homogenous, flat half-space. Although actual volcanic sources are not embedded cavities of simple shape, we assume that these models may reproduce the stress field created by the actual magma intrusion or hydrothermal fluid injection. The dMODELS software employs a nonlinear inversion algorithm to determine the best-fit parameters for the deformation source by searching the minimum the cost function 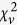 (chi square per degrees of freedom):


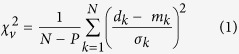


where *N* is the number of data points, *P* the number of model parameters, 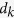 are the experimental data, 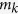 the modeling results, and 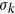 the data uncertainties. The non-linear inversion algorithm is a combination of local optimization (interior-point method^45^) and random search. This approach is more efficient for hyper-parameter optimization than trials on a grid^46^. See also Fig. 4, Supplementary Fig. S4, Supplementary Tables S3 and S4.

We tested four source geometries: a spherical source^47^, a prolate spheroid^48^, a horizontal penny-shaped source^49^ and a dike^50^ all in an elastic, homogeneous, isotropic half-space. The details of the models obtained by inversion of the data are listed in Supplementary Tables S3 and S4.

Finally, we compared the proposed models by performing F-tests on the residual 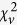 to determine if the reduction in 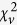 is greater than it would be expected simply because additional model parameters were added^51^ – Supplementary Table S3.

## Additional Information

**How to cite this article**: Di Vito, M. A. *et al*. Magma transfer at Campi Flegrei caldera (Italy) before the 1538 AD eruption. *Sci. Rep.* **6**, 32245; doi: 10.1038/srep32245 (2016).

## Supplementary Material

Supplementary Information
