# Supplementary material for: Magma transfer at Campi Flegrei caldera (Italy) before the 1538 AD eruption
Source: Sci Rep. 2016 Aug 25;6:32245. doi: 10.1038/srep32245 (PMC4997606; doi:10.1038/srep32245)
Supplement: Supplementary Information [file srep32245-s1.pdf]

Magma transfer at Campi Flegrei caldera (Italy) before the 1538 AD eruption

Mauro A. Di Vito[1]*, Valerio Acocella[2], Giuseppe Aiello[3], Diana Barra[1,3], Maurizio Battaglia[4,5], Antonio Carandente[1], Carlo Del Gaudio[1], Sandro de Vita S.[1], Giovanni P. Ricciardi[1], Ciro Ricco[1], Roberto Scandone[2], Filippo Terrasi[6]

SUPPLEMENTARY INFORMATION

The Supplementary Information consists of:

a) Example of reconstruction of the ground movements at Capo Miseno site;
b) Supplementary Table S1. Ground deformation at 20 sites along the coastline of Campi Flegrei;
c) Supplementary Table S2. Summary of the methods, data and references used in the reconstruction of ground deformation for each analyzed period and site;
d) Supplementary Tables 3a and 3b;
e) Supplementary Tables 4a and 4b;
f) Information on the inversion of the tilt data;
g) Further references for Supplementary Tables S1 and S2.

a) Example of the reconstruction of the vertical ground movements using a multidisciplinary approach at the Capo Miseno site

The Capo Miseno site (Benchmark 58) includes a pillar of the entrance to the Miseno Roman military harbour (dated to 12 BC), a 1st century AD fishpond used until the 4th century, and a Roman bath house (*therma*), used for producing clay pots and still active during the 6th century. Later, the area was invaded by the sea because of ground subsidence (Supplementary Fig. S1 and Tab. S1) until the beginning of the 12th century.
The starting date of our analysis is 35±2 BC, also used as a reference for the sea level in all the reconstructed sites, corresponding to the construction of many coastal Roman structures (roads, buildings, harbours) during the reign of Augustus.
The therma, located 11 m above the 35 BC sea level, was invaded by the sea only in the 6th century AD, as testified by the marine sedimentation inside the edifice and above the 6th century kiln. The thickness of marine sediments is 2.5 m, indicating that the sea reached at

least the level B (Supplementary Fig. S1a), 14 m above the sea level in 35 BC. The palaeontological analysis of the sediments has revealed a benthic foraminifera assemblage including the species *Peneroplis pertusus*, and, subordinately, *Adelosina longirostra*, *Ammonia parkinsoniana, Bolivina spathulata, Elphidium crispum, E. complanatum, Lobatula lobatula, Massilina secans, Planorbulina mediterranensis, Quinqueloculina ungeriana* and *Siphonaperta aspera*, indicating a sedimentation in a marine infralittoral paleoenvironment and implying the submergence of the entire area up to level B, 2.5 m above the *therma* floor (Supplementary Fig. S1a). Marine sedimentation in the *therma* ended in the 11-12th century, as shown by the age of an archaeologically well-dated new kiln built on top of the marine sequence. AMS ^{14}C dates corroborate our reconstruction (Supplementary Figure S1a).

The difference of altitude between the sea-level at the time of the construction of the harbour (level A) and the top of the marine sequence filling the therma (level B) permits to define that the area of Capo Miseno subsided of at least 14 m from the 1st century BC until the beginning of the 12th century (Supplementary Fig. S1a). The fishpond, built after the harbour, when the relative sea level was already higher than level A and abandoned since the 4th century AD, indicates that the submersion was progressive. Its elevation permits to demonstrate that the sea level at that time was already raised of about 5 m [52].

The subsequent net uplift from 1251 to 1538 is ~6.8 m. This is indicated by the difference between the altitude of the top of the marine sequence (level B) and the sea level in 1905 (level H) (Supplementary Fig. S1a), knowing that there was no uplift between 1538 and 1905 (Fig. 2) [10]. An analysis of historical images of landscape and sources (Supplementary Figures S2, S3a and S3b – see Supplementary Tables 1 and 2 for references) has yielded a detailed picture of the major uplift variations in the 1251 – 1538 time interval; the largest uplift in the Miseno area occurred between 1251±50 and 1536±1 (approximately 6 m), while between 1536 and the beginning of the eruption (September 1538) the uplift was approximately 1 m.

In particular, using the [18] description, it is possible to define that the top of the coastal structures between Baia and Miseno (pillars and coastal roads) was at sea-level in 1536 (between levels E and F of Supplementary Figure S1a).

In fact, in his description of the landscape in 1536, Alberti [18] says:

"... *le acque marine hanno poi ogni cosa quivi sommerso insieme con la via Silicata di Selci (come insino ad hora essendo il mare chiaro in più luoghi si vede) la quale è rimasta sotto l'acque coperta ... seguitando più oltre, lungo il lito verso Baie, appaiono parte de sontuosi edifici sopra l'alte rupi, le quali scendono giù al lito, edificati sopra gli alti, et grossi Piloni,*

che ora sono nell'acque marine. ... Erano posti detti piloni con gli edifici sopra la strada, per la quale si caminava a Baie dalle habitationi poste circa il mare Morto, ma ora vedonsi i fondamenti et etiandio parte di dette fabriche co i piloni nel mare, con la bella strada di selci silicata, come dissi".

"… then marine water submerged everything, along with the Silicata di Selci road (as till now it is possible to see, thanks to the clarity of the water), which remained covered by the sea… carrying on down, along the coast toward Baia, the remnant of sumptuous buildings appear along the slopes that shelve to the sea, just on top of the big Pillars, which presently stay into the water. …These pillars and buildings were placed above the road from the Mare Morto houses to Baia, but now their foundations and part of them, with the pillars, stay into the sea, as well as the nice paving-stone road, as I mentioned before"

The Author well describes the road between Baia and the Miseno harbour (*mare Morto auct.*) that continued upon the breakwater pillars of the Miseno military harbour. These pillars, although visible, were still underwater in 1536.

The details of the accurate maps of Supplementary Figures S2 and S3 corroborate our reconstruction. In fact, in the [53] image (Supplementary Fig. S2), which is younger than 1515-1520 and predates the Monte Nuovo eruption the pillars and the coastal roads between Baia and Miseno are not reported, and therefore were still submerged, while only the reported entrance of the Miseno military harbour and the connected bridge were above sea level (4 in Fig. S2 and S3a). According to the Alberti [18] description this condition lasted till 1536. The emersion of the coastal structures, well documented in Figures S3a and b (both showing the area soon after the Monte Nuovo eruption), indicates the further ground uplift of about 1 m, occurred between 1536 and 1538.

According to the chronicles describing the Monte Nuovo eruption, the faster uplift immediately preceding and accompanying the eruption did not significantly affect the Miseno area (see Supplementary Table S1 for reference); the amount of any sin-eruptive deformation in the Miseno area is included in the estimated error of 0.5 m. Furthermore, no significant deformation has been detected at Capo Miseno from 1539 to the present (Supplementary Figure S1b).

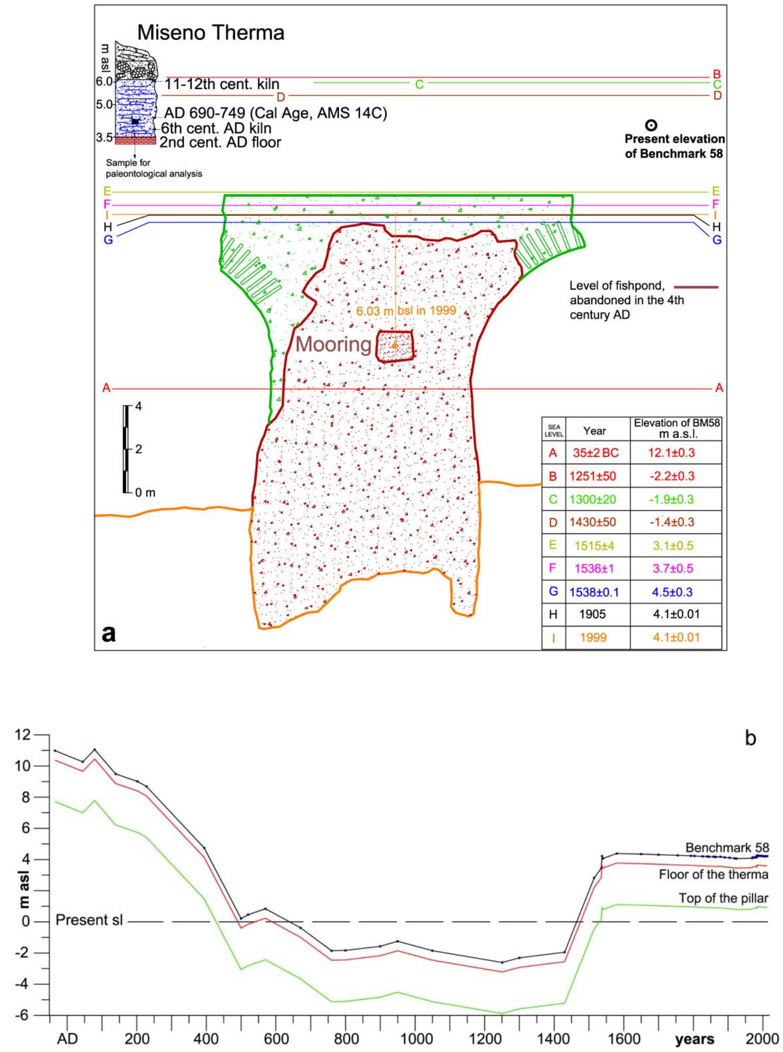

***Supplementary Figure S1**- Reconstruction of the ground movements in the Miseno site. (**a**) Sketch of a pillar of the Roman naval base and stratigraphic section of a bath complex (upper left corner) localized inland, close to benchmark 58 (Fig. 1). In brown the existing structures and in green the supposed shape of the pillar during Roman time. Elevation measurements refer to the nearby benchmark n. 58 (BM58) of the levelling network of the Osservatorio Vesuviano. The position of sea level (lines A to I) has been reconstructed from 35±2 BC (level A) to 1999 (level I) using geological, archaeological, historical, paleontological, geochronological and geodetic data. **b**) Diagram showing the reconstruction of the ground movements in the last 2000 years at benchmark 58 (black line, measured with precise levelling techniques in the last century), at the floor of the Miseno therma (red line) and at the top of the pillar of the military harbour of Miseno (green line, destroyed in 1943). The initial height differences between the three reference features (benchmark, therma and pillar) allows their time of emersion to be inferred in detail. Present sl=present sea level.*

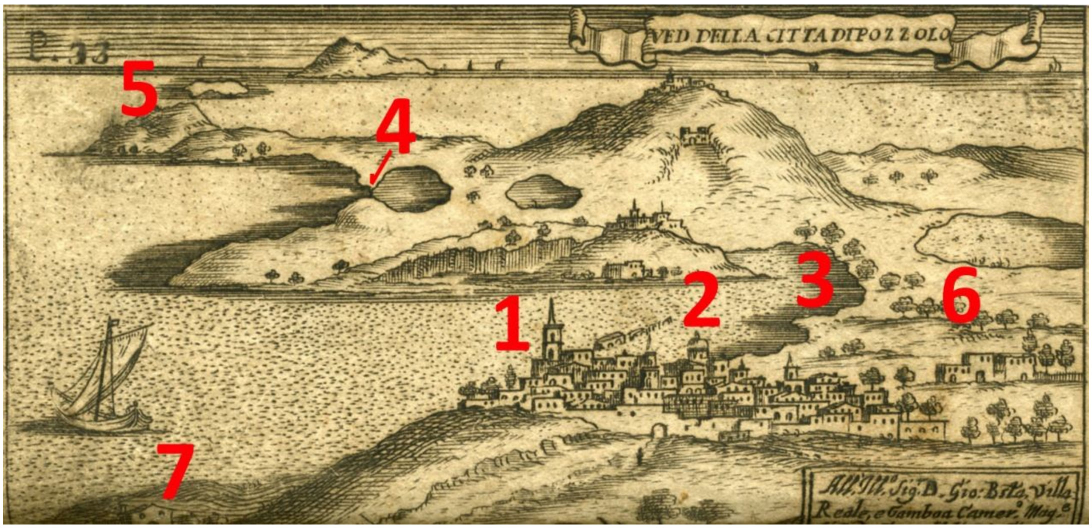

Supplementary Figure S2. *The Pozzuoli and Capo Miseno landscape before Monte Nuovo in an early 16th century print by an unknown author, reported by [53]. In particular, the presence of the Dominican monastery and the absence of Mt. Nuovo allows this print to be dated between 1515-1520 (foundation of the monastery) and the Mt. Nuovo eruption. 1: The centre of Pozzuoli (Rione Terra); 2: the Caligola pier, largely emerged from the sea; 3: Punta Caruso, part of the Roman commercial harbour (Portus Julius), emerged before the eruption; 4: entrance of the Miseno Roman military harbour with the bridge (indicated by the red arrow); 5: Capo Miseno; 6: the area of the eruption, without the Monte Nuovo volcanic edifice; 7: the Dominican monastery.*

INGV – Osservatorio Vesuviano library.

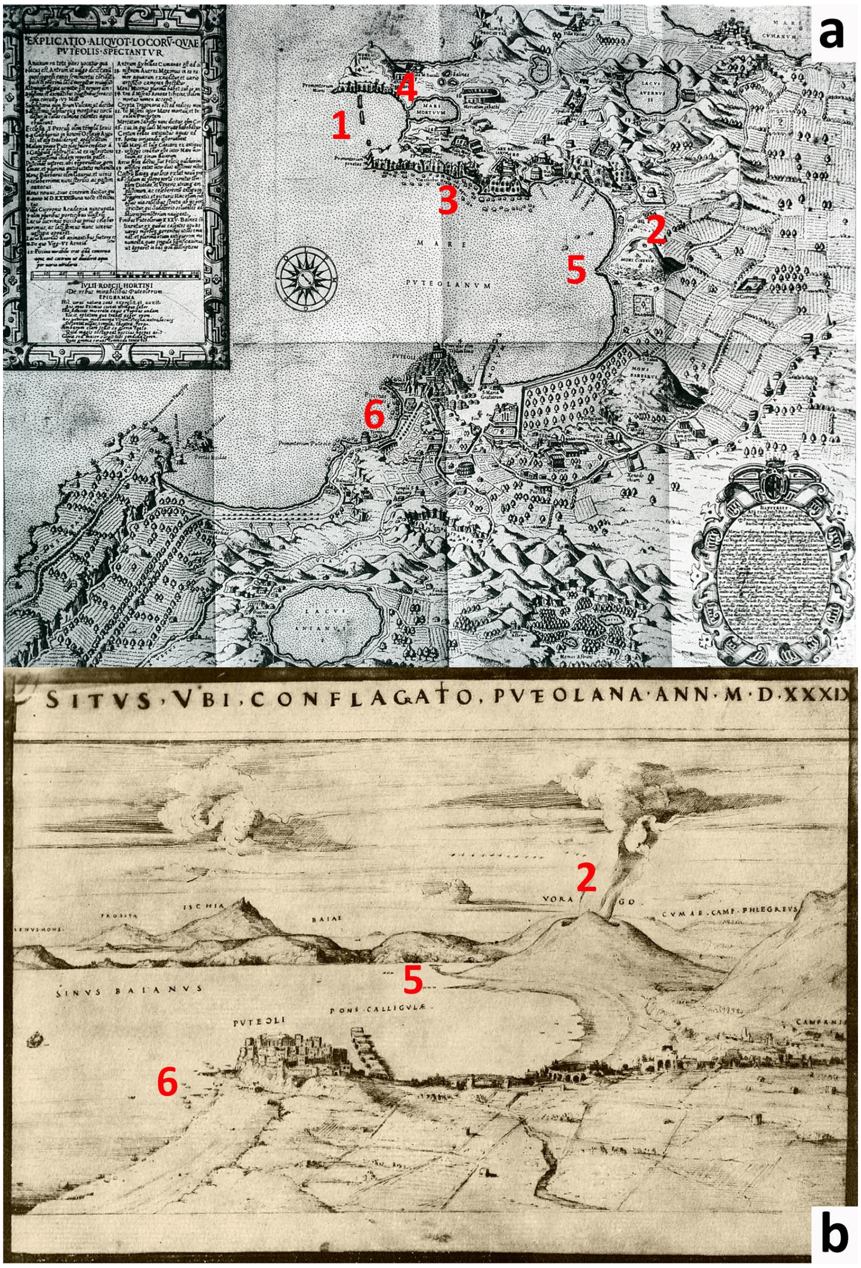

Supplementary Figure S3*. *a) *Cartaro [20], map of Campi Flegrei coastal area (Ager Puteolanus) after the Monte Nuovo eruption, showing the emerged Roman remains, which allowed estimation of the local pre-eruptive uplift. 1: breakwater pillars of the Miseno military harbour, still underwater in the [53] image (Supplementary Figure S2); 2: the Monte Nuovo cone; 3 and 6: Baia and Pozzuoli fishponds, and other coastal remains still submerged in the [53] image; 4: entrance of the Miseno military harbour with the bridge; 5: Portus Julius pillars in the Punta Caruso offshore, still underwater in the [53] image.* ***b)*** *De Hollanda [19], map of Campi Flegrei in 1539. Numbers as in Supplementary Figure S3a.*
S3a: INGV – Osservatorio Vesuviano library; S3b: Marco Besso library, Rome.

b) Supplementary Table S1 – Ground deformation at 20 sites along the coastline of Campi Flegrei. The data have been obtained through a critical analysis of sources (references and sources column) and their integration with field data and laboratory analyses.

Bench-mark	Site	Coord UTM WGS 84 (E) (N)	Uplift (m) 1251±50 – 1400	Uplift (m) 1400 - 1536	Uplift (m) 1536 - 1538	Total deformation (m) 1251±50 – 1538	Used references and sources
**58**	**Miseno harbour and thermae**	422865 4515494	0.7±0.5	5.3±0.8	0.8±0.5	6.8	[18,19,20,21,52,53,56, 57,58,72,75]
**49**	**Baia castle**	422265 4518084	0.7±0.5	6.0±0.5	1.8±0.5	8.5	[18,19,20,21,53,58,70, 72,75]
**44**	**Mercurio thermae**	421682 4519025	0.6±0.5	4.5±0.5	1.1±0.5	6.3	[18,19,20,21,53,58,60, 61,62,63,64,65,66,70, 75]
**43A**	**Baia**	421725 4519312	0.7±0.5	6.0±0.5	1.1±0.5	6.2	[18,19,20,21,53,54,55, 58,60,61,62,63,64,65, 66,70,75]
**42**	**Punta Epitaffio**	422108 4519491	0.6±0.5	5.1±0.8	1.8±0.5	7.5	[18,19,20,21,53,54,55, 58,60,61,62,63,64,65, 66,70,75]
**Ninfeo**	**Ninfeo**	422185 4519426	0.6±0.5	5.3±0.5	4.1±0.5	10.1	[18,20,21,53,58,62,63, 64,65,66,75]
**41**	**Lucrino**	422153 4519831	0.7±0.5	5.0±0.5	5.1±0.5	10.7	[18,19,20,21,53,54,55, 62,63,64,65,66,72,75]
**39A**	**Lucrino 1**	422758 4520259	0.7±0.5	5.5±0.8	13.2±1.5	19.3	[18,19,20,21,53,54,55, 62,63,64,65,66,72,75]
**37**	**Portus Julius**	423627 4520568	0.7±0.5	6.1±0.5	18.8±2.5	25.6	[19,20,21,53,54,55,60, 61,62,63,64,65,66,67, 72,75]
**124**	**Apollo temple**	423054 4521512	0.5±0.5	4.0±0.5	11.5±2.0	16.0	[19,20,21,53,58,70,72, 75]
**35A**	**Ripa Puteolana**	424241 4520607	0.9±0.5	7.1±0.5	7.9±0.5	15.9	[19,20,21,62,63,64,65, 66,67,70,72,75]
**30**	**Serapeo**	425803 4519803	1.4±0.5	10.4±0.8	2.4±0.5	14.3	[10,15,16,19,20,53,59, 60,61,68,69,70,71,74, 75]
**FPOZ**	**Molo Caligoliano 15th Pillar**	425261 4519320	1.5±0.5	12.3±0.8	3.1±0.5	16.9	[15,16,19,20,21,22,59, 60,61,68,69,72,73,74, 75]
**X Pila**	**Molo Caligoliano 10th Pillar**	425389 4519342	1.5±0.5	12.3±0.8	3.0±0.5	16.9	[15,16,19,20,21,22,59, 60,61,68,69,72,73,74, 75]
**28**	**St. Maria delle Grazie**	425698 4519496	1.5±0.5	11.9±0.8	2.7±0.5	16.0	[15,16,19,20,21,60,61, 68,70,75]
**25A**	**Corso Umberto**	426301 4519308	1.5±0.5	12.2±0.8	2.2±0.5	15.9	[15,16,19,20,22,69,72, 74,75]
**22**	**Via Napoli**	427231 4518951	1.3±0.5	11.0±1.0	1.3±0.5	13.5	[15,16,19,20,53,72,75]
**17A**	**La Pietra**	428822 4518927	0.9±0.5	7.0±0.8	0.4±0.5	8.3	[19,20,70,72,75]
**14**	**Bagnoli**	429831 4518474	0.6±0.5	5.1±0.5	0.3±0.5	6.0	[20,70,72,75]
**194**	**Molo di Nisida pillar**	429732 4516510	0.5±0.5	4.2±0.5	0.2±0.5	4.9	[20,59,60,61,62,75]

c) Supplementary Table S2. Summary of the methods, data and references used in the reconstruction of ground deformation for each analyzed period and site.

Bench mark	Site	Year	Method and related data	Refe-rences
58	Miseno harbour and thermae	1400	Reconstruction of the coastline on the basis of the definitive emersion of the Miseno Therma. Evaluation of the difference in elevation between the Therma and the manmade structures along the coastline. Definition of the position and height of emerging baths that were previously submerged. Surface and subsurface geological data (stratigraphical, sedimentological, palaeontological, archaeological data).	[52,56,57, 58,72,75, present paper]
		1536	Reconstruction, using historical maps and chronicles, of the location and height of the following manmade Roman coastal features: roads, pillars, bridge, harbour structures.	[18,21,53, 75]
		1538	Reconstruction of the vertical ground movements based on the variation in elevation of the marine and coastal sediments filling the Miseno Therma and the sea level (sl) in 1251. Evaluation using historical maps of the position of manmade coastal structures that were submerged until 1536.	[19,20,75]
49	Baia castle	1400	Reconstruction of the coastline on the basis of the position of the notch along the Baia sea-cliff cutting Roman walls and buildings. Correlation of notches along the coast with marine sediments and mollusc concretions between Miseno and Punta Epitaffio. Evaluation of the ground movements using historical images and descriptions of active Roman baths.	[58,70,72, 75]
		1536	Reconstruction, using historical maps and chronicles, of the position of the following manmade Roman coastal features: roads; pillars; harbour structures, fishponds.	[18,21,53, 75, present paper]
		1538	Reconstruction of the vertical ground movements based on the variation in elevation of notches and the sl in 1251. Evaluation on historical maps of the position of manmade coastal structures that were submerged until 1536.	[19,20,75]
44	Mercurio thermae	1400	Reconstruction of the coastline on the basis of the position of the notch cutting the Therma walls. Evaluation of the vertical ground movements on the basis of historical images and descriptions of the use of the Roman baths.	[58, 70,75]
		1536	Reconstruction, using historical maps and chronicles, of the position of the following manmade Roman coastal features: roads; pillars; harbour structures, fishponds.	[18, 21,53,58, 75]
		1538	Reconstruction of the vertical ground movements based on the variation in elevation of notch and the sl in 1251. Evaluation using historical maps of the position of manmade coastal structures that were submerged until 1536.	[19,20,60, 61,62,63, 64,65,66, 75]
43A	Baia	1400	No data. Reconstruction of the coastline on the basis of the nearest sites (44 and 42).	-
		1536	Reconstruction, using historical maps and chronicles, of the position of the following manmade Roman coastal features: roads, pillars, harbour structures, fishponds.	[18, 21,53,58, 75]
		1538	Reconstruction of the vertical ground movements based on the variation in elevation between 1536 and 1538, using historical maps and chronicles reporting and describing the relative position of manmade coastal structures.	[19,20,60, 61,62,63, 64,65,66, 75]

Supplementary Table S2 (continued)

Bench mark	Site	Year	Method and related data	Refe-rences
42	Punta Epitaffio	1400	Reconstruction of the coastline on the basis of the position of the notch cutting the Roman structures (thermae, Portus Julius, Sinus Baianus). Evaluation of the vertical ground movements on the basis of historical images and descriptions of the use of Roman baths.	[54,55,58, 70,75, present paper]
		1536	Reconstruction, using historical maps and chronicles, of the position of the following manmade Roman coastal features: roads, pillars, harbour structures, fishponds.	[18, 21,53,75]
		1538	Reconstruction of the vertical ground movements based on the variation in elevation of notch and the sl in 1251. Evaluation using historical maps and chronicles of the eruption of the position of manmade coastal structures, submerged until 1536.	[19,20,60, 61,62,63, 64,65,66, 75]
Ninfeo	Ninfeo	1400	Roman structure partially submerged at time of its construction, used to define the coastline in Roman times. The 1400 sl was reconstructed on the basis of the nearest benchmark (42).	-
		1536	Reconstruction, using historical maps and chronicles, of the position of the Roman coastal roads that was described as being at sl.	[18, 21,53,58, 75]
		1538	Reconstruction of the vertical ground movements based on historical maps and chronicles. describing the marine area around Ninfeo.	[20,58,62, 63,64,65, 66,75]
41	Lucrino	1400	Reconstruction of the coastline on the basis of the position of the manmade Roman structures (thermae, Portus Julius and pillars, Sinus Lucrinus). Evaluation of the ground movements using historical images and descriptions .	[54,55,72, 75]
		1536	Reconstruction, using historical maps and chronicles, of the position of the Portus Julius pillars.	[18,21,53, 75]
		1538	Reconstruction of the vertical ground movements based on historical maps and chronicles of the eruption defining the position of manmade coastal structures that were submerged until 1536.	[19,20,62, 63,64,65, 66,75]
39A	Lucrino 1	1400	Harbour in Roman times. The reconstruction was carried out taking into account the thickness of the Monte Nuovo products whose base overlies shallow-sea sediments. Evaluation of the ground movements using historical images and descriptions . Surface and subsurface geological data (stratigraphical, sedimentological, palaeontological, archaeological data).	[53,54,55, 72,75,present paper]
		1536	Reconstruction of the coastline using historical maps and chronicles defining the position respect to the coastline of the Roman road, pillars and harbour structures.	[18,21,53, 75]
		1538	Reconstruction of the vertical ground movements based on historical maps and chronicles of the eruption defining the position of manmade coastal structures and of morphological coastline features. Reconstruction also based on the variation in elevation of the shallow-sea sediments.	[19,20,62, 63,64,65, 66,75]

Supplementary Table S2 (continued)

Bench mark	Site	Year	Method and related data	Refe-rences
37	Portus Julius	1400	Reconstruction of the coastline on the basis of the position of beach and lacustrine sediments overlying Roman structures built on land. The reconstruction takes into account the thickness of the Monte Nuovo products. Evaluation of the ground movements using historical images and descriptions. Surface and subsurface geological data (stratigraphical, sedimentological, palaeontological, geochronological, archaeological data).	[54,55,70, 72,75,present paper]
		1536	Reconstruction of the coastline using historical maps and chronicles defining the position of the Roman road, pillars and harbour structures.	[21,53,75]
		1538	Reconstruction of the vertical ground movements based on historical maps and chronicles of the eruption that define well the position of manmade coastal structures and of the morphological coastline features. Reconstruction also based on the variation in elevation of the shallow-sea sediments.	[19,20,60, 61,62,63, 64,65,66, 67,75]
124	Apollo temple	1400	Reconstruction of the coastline on the basis of presence and elevation of lagoon sediments covering the Apollo temple floor. Evaluation of the ground movements using historical images and descriptions.	[58,70,72, 75]
		1536	The sl position was inferred using the benchmarks 39A and 37 and using the historical maps of the Lucrino coastline.	[21,53,72, 75]
		1538	Reconstruction of the vertical ground movements using a detailed historical map.	[19,20,75]
35A	Ripa Puteolana	1400	Reconstruction of the coastline on the basis of the position of beach and lagoon sediments overlaying Roman structures built on land and covered by the Monte Nuovo products. Evaluation of the ground movements using historical images and descriptions. Surface and subsurface geological data (stratigraphical, sedimentological, palaeontological, archaeological data).	[70,72,75, present paper]
		1536	Reconstruction of the coastline using historical maps and chronicles defining the position of the Roman road, pillars and harbour structures.	[21,75]
		1538	Reconstruction of the vertical ground movements based on historical maps and chronicles of the eruption that define well the position of manmade coastal structures and of the morphological coastline features. Reconstruction also based on the variation in elevation of the shallow-sea sediments.	[19,20,62, 63,64,65, 66,67,72, 75]
30	Serapeo	1400	Reconstruction of the coastline based on the position of geomorphological features and manmade structures at well defined elevations (Serapeo lithodome holes, coastal buildings, still submerged thermal bath structures). Also based on urban development, historical, cartographical, palaeontological, geochronological and archaeological data. The age of extinction of the mollusc colonies on the Serapeo columns and the Rione Terra sea-cliff was used to define the definitive emersion of the area.	[15,16,59, 69,70,75]
		1536	Definition of the position of manmade Roman coastal structures and reconstruction of their vertical movements using the position of Roman roads, pillars, buildings, harbour structures. The reconstruction was based on historical maps and descriptions, urban expansion of the town, emergence of thermal baths and Royal edicts.	[15,16,53, 59,74,75]
		1538	Reconstruction of the vertical ground movements based on historical maps and chronicles that define well the position of manmade coastal structures and of the coastline features. Reconstruction also based on the variation in elevation of the lithodome holes.	[10,15,16, 19,20,59, 60,61,68, 71,75]

Supplementary Table S2 (continued)

Bench mark	Site	Year	Method and related data	Refe-rences
FPOZ	Molo Caligoliano 15th Pillar	1400	Reconstruction of the coastline based on the position of geomorphological features and harbour structures. Also based on urban development and historical data.	[15,16,59, 69,75]
		1536	Definition of the coastline on the basis of the position of pillars, buildings and harbour structures. The reconstruction was based on historical maps and descriptions, urban expansion of the town and Royal edicts.	[15,16,59, 72,74,75]
		1538	Reconstruction of the coastline based on historical maps reporting the manmade coastal and harbour structures.	[15,16,19, 20,21,22, 59,60,61, 68,69,73, 75]
X Pila	Molo Caligoliano 10th Pillar	1400	Reconstruction of the coastline based on the position of geomorphological features and harbour structures. Also based on urban development and historical data.	[15,16,59, 69,75]
		1536	Definition of the coastline on the basis of the position of pillars, buildings and harbour structures. The reconstruction was based on historical maps and descriptions, urban expansion of the town and Royal edicts.	[15,16,59, 72,74,75]
		1538	Reconstruction of the coastline based on historical maps that show well the manmade coastal and harbour structures.	[15,16,19, 20,21,22, 59,60,61, 68,69,73, 75]
28	St. Maria delle Grazie	1400	Reconstruction of the coastline based on urban expansion of the town toward the sea. Evaluation of the ground movements based on historical images.	[15,16,70, 75]
		1536	Definition of the coastline on the basis of the construction of churches in areas previously submerged (St. Maria delle Grazie and Benedictine monastery).	[15,16,75]
		1538	Reconstruction of the coastline based on historical maps that show well the manmade coastal and harbour structures.	[15,16,19, 20,21,60, 61,68,75]
25A	Corso Umberto	1400	Reconstruction of the coastline based on the position of fossils along the Rione Terra sea-cliff. Also based on urban development, historical and cartographical data.	[15,16,69, 72,75]
		1536	Definition of the position of manmade Roman coastal structures and reconstruction of their vertical movements using historical maps and descriptions, urban expansion of the town and Royal edicts.	[15,16,22, 72,74,75]
		1538	Reconstruction of the coastline and of the vertical movements using historical maps, position and emersion of fishponds and other manmade coastal remains.	[15,16,19, 20,69,72, 75]
22	Via Napoli	1400	Reconstruction of the coastline based on the urban development of the town, historical and cartographical data.	[15,16,72, 75]
		1536	Reconstruction of the coastline and definition of vertical movements using historical maps and descriptions, urban expansion of the town and Royal edicts.	[15,16,53, 72,75]
		1538	Reconstruction of the coastline and of the vertical movements on the basis of historical maps, position and emersion of fishponds and other coastal remains.	[15,16,19, 20,53,72, 75]

Supplementary Table S2 (continued)

Bench mark	Site	Year	Method and related data	Refe-rences
17A	La Pietra	1400	Estimate of vertical displacement reconstructing the maximum sea ingression on the basis of the position of the Medieval thermal baths relative to the coastline, as deduced from historical images.	[70,72,75]
		1536	No data. The vertical displacement was estimated as a fraction of the maximum deformation recorded in 1538.	-
		1538	Reconstruction of the coastline and of the vertical movements using historical maps, position and emersion of fishponds and other coastal remains.	[19,20,75],
14	Bagnoli	1400	Estimate of vertical displacement reconstructing the maximum sea ingression on the basis of the position of the Medieval thermal baths relative to the coastline, as deduced from historical images.	[70,72,75]
		1536	No data. The vertical displacement was estimated as a fraction of the maximum deformation recorded in 1538.	-
		1538	Reconstruction of the coastline and of the vertical movements on the basis of a historical map.	[20]
194	Molo di Nisida pillar	1400	Estimation of vertical displacement on the basis of morphological evidence.	[59,60,61, 75]
		1536	No data. The vertical displacement was estimated as a fraction of the maximum deformation recorded in 1538.	-
		1538	Reconstruction of the coastline and of the vertical movements on the basis of historical maps.	[20,60,61, 62,75]

d) Supplementary Table 3a - Summary of inversion results of the historical deformation at Campi Flegrei. Best fit solution is in bold (see also Fig. 4 and Suppl. Table S4)

1400 - 1536 (uplift)

Source	χ^2_v	X [km] (*)	Y [km] (*)	depth [km]	ΔV [km^3]	U [m]	dip (°)	strike (°)	length [km]	width [km]	Radius [km]	A
Opening crack	0.71	-1.6<br>1.8	-3.7<br>-3.4	0.1<br>5.4	4.59	246	81	-6	3.5	5.4		
Sill-like	0.78	-0.1	-1.9	5.5	0.93						0.8	
**Sphere**	**0.64**	**-0.1**	**-1.9**	**3.9**	**0.95**						**1.0**	
Spheroid	0.61	-0.2	-0.9	4.0	0.95		40	170			1.0	0.60

1536-1538 - deeper source (uplift + tilt)

Source	χ^2_v	X [km] (*)	Y [km] (*)	depth [km]	ΔV [km^3]	U [m]	dip (°)	strike (°)	length [km]	width [km]	Radius [km]	A
Opening crack	1.66	-6.4<br>-3.6	2.3<br>5.0	0.9<br>4.1	0.23	9	30	-110	3.9	6.5		
Sill-like	2.13	-2.7	1.1	4.4	0.21						0.5	
**Sphere**	**1.99**	**-2.7**	**1.2**	**3.2**	**0.23**						**0.5**	
Spheroid	3.07	-2.4	1.2	4.2	0.34		79	-95				0.99

1536-1538 - shallower source (residual uplift)

Source	χ^2_v	X [km] (*)	Y [km] (*)	depth [km]	ΔV [km^3]	U [m]	dip (°)	strike (°)	length [km]	width [km]	Radius [km]	A
Opening crack	2.96	-1.9<br>-2.3	0.1<br>-0.1	0.4<br>40.0	4.46	258	78	-156	0.4	40.4		
**Sill-like**	**2.94**	**-2.6**	**0.9**	**0.1**	**0.03**						**1.5**	
Sphere	3.77	-2.5	1.0	0.6	0.05						0.5	
Spheroid	4.35	-2.6	1.0	0.5	0.04		22	-121				0.28

(*) bm 30 Serapeo (425803, 4519803) UTM

d) Supplementary Table S3b - F-test

1400-1536 (uplift)

Source	χ^2_v	P	*v*	*N*	F_{exp}	$F_{th\ (1\%)}$
Sphere	0.64	4	16	20	1.3	5.7
Spheroid	0.61	7	13	20		

1536-1538 - deeper source (tilt + uplift)

Source	χ^2_v	P	*v*	*N*	F_{exp}	$F_{th\ (1\%)}$
Sphere	1.99	4	40	44	3.0	3.9
Opening crack	1.66	8	36	44		

1536-1538 - deeper source (tilt + uplift)

Source	χ^2_v	P	*v*	*N*	F_{exp}	$F_{th\ (1\%)}$
Sphere	3.77	4	50	54	15.1	7.2
Sill	2.94	5	49	54		

e) Supplementary Table S4a - Site by site comparison between the uplift and the best fit solution for 1400-1536 (see also Fig. 4 and Supplementary Table S3)

BM	site	X [UTM]	Y [UTM]	L (km)	uplift [m]	error	model
194	Nisida	429732	4516510	0.0	4.2	0.5	4.6
14	Bagnoli	429831	4518474	2.5	5.1	0.5	4.8
17A	LaPietra	428822	4518927	3.6	7.0	0.8	6.7
22	ViaNapoli	427231	4518951	5.2	11.0	1.0	11.0
25A	CorsoUmberto	426301	4519308	6.3	12.2	0.8	12.0
28	SMariaGrazie	425698	4519496	6.9	11.9	0.8	11.8
XPila	MoloCalig10	425389	4519342	7.3	12.3	0.8	12.2
FPOZ	MoloCalig15	425261	4519320	7.4	12.3	0.8	12.1
30	Serapeo	425803	4519803	8.2	10.4	0.8	10.8
35A	RipaPuteolana	424241	4520607	10.2	7.1	0.5	7.2
124	ApolloTemple	423054	4521512	11.7	4.0	0.5	4.2
37	PortusJulius	423627	4520568	12.8	6.1	0.5	6.4
39A	Lucrino1	422758	4520259	13.7	5.5	0.8	5.5
Ninfeo	Ninfeo	422185	4519426	14.9	5.3	0.5	5.4
41	Lucrino	422153	4519831	15.3	5.0	0.5	5.0
42	PuntaEpitaffio	422108	4519491	15.6	5.1	0.8	5.2
43A	Baia	421725	4519312	16.0	6.0	0.5	4.6
44	MercurioThermae	421682	4519025	16.3	4.5	0.5	4.7
49	Castello	422265	4518084	17.5	6.0	0.5	6.2
58	MisenoTerme	422865	4515494	20.5	5.3	0.8	5.6

e) Supplementary Table S4b - site by site comparison between the uplift and the best fit solution for 1536-1538 (see also Fig. 4 and Supplementary Table S3)

BM	site	X [UTM]	Y [UTM]	L (km)	uplift [m]	error	deep	shallow	deep + shallow
194	Nisida	429732	4516510	0	0.2	0.5	0.3	0.0	0.3
14	Bagnoli	429831	4518474	2.517	0.3	0.5	0.4	0.0	0.4
17A	LaPietra	428822	4518927	3.629	0.4	0.5	0.5	0.0	0.5
22	ViaNapoli	427231	4518951	5.233	1.3	0.5	1.0	0.0	1.0
25	CorsoUmberto	426301	4519308	6.254	2.2	0.5	1.6	0.1	1.7
28	SMariaGrazie	425698	4519496	6.923	2.7	0.5	2.1	0.1	2.2
XPila	MoloCalig10	425389	4519342	7.268	3.0	0.5	2.3	0.2	2.5
FPOZ	MoloCalig15	425261	4519320	7.398	3.1	0.5	2.4	0.2	2.6
30	Serapeo	425803	4519803	8.232	2.4	0.5	2.1	0.1	2.2
35A	RipaPuteolana	424241	4520607	10.179	7.9	0.5	4.4	3.2	7.6
124	ApolloTemple	423054	4521512	11.703	11.5	2.0	5.1	6.3	11.4
37A	PortusJulius	423627	4520568	12.807	18.8	2.5	5.0	13.9	18.9
39A	Lucrino1	422758	4520259	13.729	13.2	1.5	4.8	7.1	11.9
Ninfeo	Ninfeo	422185	4519426	14.870	4.1	0.5	3.4	0.4	3.8
41	Lucrino	422153	4519831	15.276	5.1	0.5	3.9	1.0	4.9
42	PuntaEpitaffio	422108	4519491	15.619	1.8	0.5	3.4	0.4	3.8
43A	Baia	421725	4519312	16.042	1.1	0.5	2.9	0.2	3.1
44	MercurioThermae	421682	4519025	16.332	1.1	0.5	2.6	0.2	2.8
49	Castello	422265	4518084	17.456	1.8	0.5	2.0	0.1	2.1
58	MisenoTerme	422865	4515494	20.500	0.8	0.5	0.7	0.0	0.7

f) Inversion of the tilt data

Tilt vectors (Supplementary Figure 4) have been calculated from the uplift measured at 17 (out of 20) selected benchmarks (BMs) (Supplementary Table 1), appropriately spaced along the coast from Miseno to Nisida. Our procedure calculates the height variation through time at the 17 BMs, normalized by the distance between them. The height variation is related to the tilt by

$$\tau(BM_x,BM_y) = [\delta\Delta h(BM_x)- \delta\Delta h(BM_y)]/L$$

where $\delta\Delta h(BM) = \Delta h(BM)t_2- \Delta h(BM)t_1$ is the change in height Δh at a given benchmark BM between the time t_1 and the time t_2, and L is the distance between two benchmarks BM_x-BM_y. The algorithm evaluates the tilt τ in m/km or mradians reiteratively among each considered BM. The mean value of each iteration gives the tilt estimate at each BM.

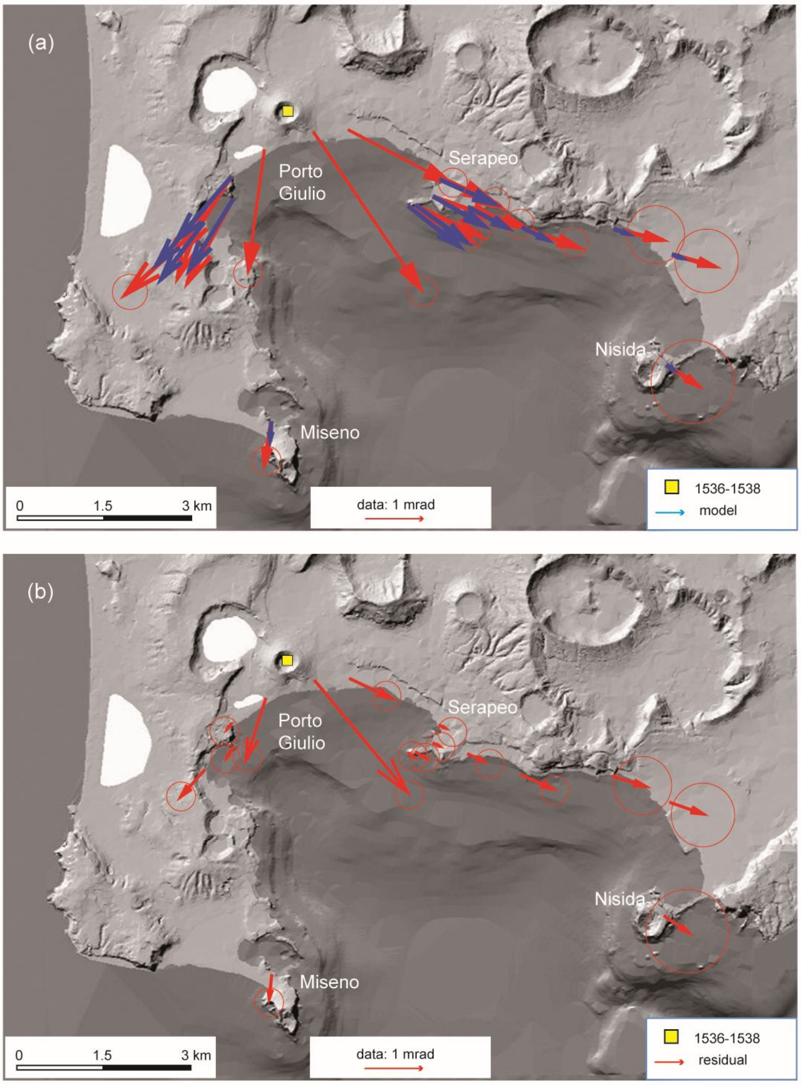

Supplementary Figure S4. *Comparison between the measured (red arrows) and modelled (blue arrows) tilt vectors for the years 1536-1538. The best fit source (orange square) has been determined by the joint inversion of uplift and tilt data and is consistent with a radially symmetrical source with a depth between 3.2 and 4.4 km and a volume change between 0.21 and 0.34 km*^{3} *(deeper source in Supplementary Table 2a). Details of the best fit sources are available in Supplementary Table 2a. Tilt ellipse errors are equivalent to the 95% confidence level.*

Digital Terrain Model by INGV – Osservatorio Vesuviano.

g) *Further references for Supplementary Tables S1 and S2*

[52] Cinque, A., Russo, F. & Pagano, M. La successione dei terreni di età post-Romana delle Terme di Miseno (Napoli): nuovi dati per la storia e la stratigrafia del Bradisisma Puteolano. *Boll. Soc. Geol. It.* **110**, 231-244 (1991)

[53] Parrino, D.A. *Nuova Guida dei Forestieri per le Antichità curiosissime di Pozzuoli*. Napoli (1727)

[54] Scherillo, G. Nota sul Porto Giulio. *Rendiconto della Reale Accademia di Archeologia e Lettere di Belle Arti,* 46-70, Napoli (1863a).

[55] Scherillo, G. Seconda Nota sul Porto Giulio*. Rendiconto della Reale Accademia di Archeologia e Lettere di Belle Arti,* 19-36, Napoli (1863b).

[56] Benini, A., Ferrari, G. & Lamagna, R. Le peschiere di Lucullo (Miseno-Napoli). Atti del Conv. Naz. di Speleologia in Cavità Artificiali. Napoli, 30 Maggio – 2 Giugno 2008. *Opera Ipogea* **1/2** (2008).

[57] Caputo, P. Attività di tutela della Soprintendenza Archeologica di Napoli e Caserta*. Atti II Conv. Naz. Archeologia subacquea*. Ministero Beni AA.CC. Roma (1989).

[58] Pagano, M. Variazioni del livello del mare fra Miseno e Baia*. Atti del Seminario internazionale: Variazioni Climatico-Ambientali e impatto sull'uomo nell'area circum–mediterranea durante l'Olocene*. Albore Livadie, C. and Ortolani, F. (Eds.), Ravello (1994).

[59] Niccolini, A. Descrizione della Gran Terma Puteolana, volgarmente detta Tempio di Serapide*. Stamperia Reale*, Napoli (1846).

[60] Parascandola, A. Il Monte Nuovo e il Lago Lucrino*. Boll. Soc. Naturalisti* **55**, 151-312 (1944-46).

[61] Parascandola, A. I fenomeni bradisismici del Serapeo di Pozzuoli. *Stabilimento Tipografico Genovese*. Napoli (1947).

[62] Marchesino, F. Copia di una Lettera di Napoli che contiene gli stupendi et gran prodigi apparsi sopra a Pozzolo. In: Parascandola, A. Il Monte Nuovo e il Lago Lucrino*. Boll. Soc. Naturalisti* **55**, 151-312 (1944-46).

[63] Porzio, S. De conflagrazione Agri Puteolani*. Simone Portii Epistola* (1538).

[64] Del Nero, F. Lettera di Francesco del Nero a Niccoló Del Benino, sul terremoto di Pozzuolo, dal quale ebbe origine la Montagna Nuova, nel 1538*. Archivio Storico Italiano* **9** (1846).
[65] Delli Falconi, M.A. Dell'incendio di Pozzuolo del MDXXXVIII*.* Napoli (1538).
[66] Rosso, G. Historia delle cose di Napoli sotto l'Imperio di Carlo V. Napoli (1545).
[67] Lusieri, G.B. Pozzuoli as seen from Monte Nuovo. *Gouache, private collection*, Napoli (1750).
[68] De Jorio, A. Guida di Pozzuoli e contorno. Napoli (1817).
[69] Morhange, C.*et al*. New data on historical relative sea level movements in Pozzuoli, Phlaegrean Fields Southern Italy. *Phys. Chem. Earth* (part A) **24**, 349-354 (1999).
[70] Pietro da Eboli. De Balneum Cantarellus. In: De Balneis Puteolanis. F. 3 v. *University Library Edimburg* (1430 circa).
[71] Hamilton, W. Campi Phlegraei - Osservazioni sui vulcani delle Due Sicilie. Napoli (1776).
[72] Ricciardi, G.P. Diario Del Monte Vesuvio. *Edizioni Scientifiche e Artistiche*, 3 vol., 893 pp. Torre del Greco (Napoli) (2009).
[73] Dubois, C. Pouzzoles Antique (Histoire et Topographie). Paris (1907).
[74] Royal Edict of King Ferdinand III, the Catholic, October 6, 1503.
[75] Istituto Idrografico della Marina. Golfo di Pozzuoli, Carta Batimetrica dai rilievi effettuati dal 1985 al 1986. Genova (1987).
